# Supplementary material for: BCG Vaccination and Mortality of COVID-19 across 173 Countries: An Ecological Study
Source: Int J Environ Res Public Health. 2020 Aug 3;17(15):5589. doi: 10.3390/ijerph17155589 (PMC7432030; doi:10.3390/ijerph17155589)
Supplement: Supplementary file 1 [file ijerph-17-05589-s001.pdf]

Supplementary Materials

BCG Vaccination and Mortalities of COVID-19 across 173 Countries: An Ecological Study

Mitsuyoshi Urashima [1,*,¶] Katharina Otani [1,2,¶], Yasutaka Hasegawa [1,3,¶], and Taisuke Akutsu [1,¶]

1 Division of Molecular Epidemiology, The Jikei University School of Medicine, Tokyo 105–8461, Japan; katharina@jikei.ac.jp (K.O.); yhearth102523@jikei.ac.jp (Y.H.); t-akutsu@jikei.ac.jp (T.A.)

2 Advanced Therapies Innovation Department, Siemens Healthcare K.K., Tokyo 141-8644, Japan; katharina.otani@siemens-healthineers.com (K.O.)

3 Hitachi,Ltd. Research & Development Group, Tokyo, 185-8601, Japan; yasutaka.hasegawa.mp@hitachi.com (Y.H.)

* Correspondence: urashima@jikei.ac.jp; Tel.: +81-3-3433-1111 (ext. 2405)

¶ These authors contributed equally to this work.

Table S1: Definition or description of each covariate.

Table S1: Definitions or description of each covariates

No	Covariates	Definition / Description / Information source	range of years	URL	access day
1	Population (n)	Elaboration of data by United Nations, Department of Economic and Social Affairs, Population Division. World Population Prospects: The 2019 Revision. (Medium-fertility variant).	2020	https://www.worldometers.info/world-population/population-by-country/	2020/05/22
2	Yearly change in population (%)				
3	Net change in population (n)				
4	Density (km2)				
5	Land area (km2)				
6	Migrants net (n)				
7	Fertility rate (n)				
8	Median age (years-~~old~~)				
9	Urban pop percentage (%)				
10	World share (%)				
11	Population between age 0 and 14 years of age (%)	United Nations Population Division, New York, World Population Prospects: The 2019 Revision; supplemented by data from the United Nations Statistics Division, New York, Demographic Yearbook 2015 and Secretariat for the Pacific	2019	https://data.un.org/_Docs/SYB/CSV/SYB62_1_201907_Population,%20Surface%20Area%20and%20Density.csv	2020/05/22
12	Population ≥ age 60 years of age (%)				
13	Population ≥ age 70 years of age (%)	Share of the population that is 70 years and older in 2015, United Nations, Department of Economic and Social Affairs, Population Division (2017), World Population Prospects: The 2017 Revision	2015	https://github.com/owid/covid-19-data/blob/master/public/data/ecdc/full_data.csv	
14	Gross domestic product (GDP) (millions US dollars)	United Nations Statistics Division, New York, National Accounts Statistics: Analysis of Main Aggregates (AMA) database, last accessed February 2018.	2017	https://data.un.org/_Docs/SYB/CSV/SYB62_230_201904_GDP%20and%20GDP%20Per%20Capita.csv	2020/05/22
15	GDP per capita (US dollars)				
16	Total unemployment (%)	International Labour Organization (ILO), Geneva, Key Indicators of the Labour Market (KILM 9th edition) and the ILOSTAT database, last accessed January 2019.	2019	https://data.un.org/_Docs/SYB/CSV/SYB62_329_201904_Labour%20Force%20and%20Unemployment.csv	2020/05/22
17	Male unemployment (%)				
18	Female unemployment (%)				
19	Labour force total participation (%)				
20	Labour force male participation (%)				
21	Labour force female participation (%)				

22	Annual incidence of tuberculosis per 100 000 population (n)	The number of new cases registered from tuberculosis in a specific year, expressed per 100,000 population, for a given country, territory, or geographic area.	2012	https://data.un.org/Data.aspx?q=tuberculosis+incidence&d=WHO&f=MEASURE_CODE%3aMDG_0000000020	2020/05/22
23	International health regulation (IHR) score (%)	Percentage of attributes of 13 core capacities that have been attained at a specific point in time. The 13 core capacities are: (1) National legislation, policy and financing; (2) Coordination and National Focal Point communications; (3) Surveillance; (4) Response; (5) Preparedness; (6) Risk communication; (7) Human resources; (8) Laboratory; (9) Points of entry; (10) Zoonotic events; (11) Food safety; (12) Chemical events; (13) Radionuclear emergencies.	2014-2019	https://www.who.int/data/gho/data/indicators/indicator-details/GHO/-average-of-13-international-health-regulations-core-capacity-scores-1st-version-of-the-questionnaire	2020/05/22
24	Universal health coverage (UHC) index	Percentage of tracer indicators in the UHC service coverage index with primary country data sources between 2013-2017	2013-2017	https://www.who.int/data/gho/data/indicators/indicator-details/GHO/data-availability-for-uhc-index-of-essential-service-coverage-(-)	2020/05/22
25	Hospital beds (n) per 10,000 population	The number of hospital beds available per every 10,000 inhabitants in a population.	2004-2015	https://www.who.int/data/gho/data/indicators/indicator-details/GHO/hospital-beds-(per-10-000-population)	2020/05/22
26	Medical doctors (n) per 10,000 population	Includes generalists, specialist medical practitioners and medical doctors not further defined, in the given national and/or subnational area. Depending on the nature of the original data source may include practising (active) physicians only or all registered physicians. The ISCO -08 codes included here are 221,2211,2212</Definition> <AssociatedTerms name="Associated terms">. The WHO framework for classifying health workers draws on the latest revisions of international classifications, including the International Standard Classification of Occupations (revision 2008), the International Standard Classification of Education (revision 2011) and the International Standard Industrial Classification of All Economic Activities (revision 4).	2001-2018	https://www.who.int/data/gho/data/indicators/indicator-details/GHO/medical-doctors-(per-10-000-population)	2020/05/22
27	Nursing midwifery (n) per 10,000 population	Number of nursing and midwifery personnel includes nursing personnel and midwifery personnel in the given national and/or subnational area. Depending on the nature of the original data source may include practicing (active) nursing and midwifery personnel only or all registered nursing and midwifery personnel The ISCO -08 codes included here are 2221,2222,3221,3222	2013-2018	https://www.who.int/data/gho/data/indicators/indicator-details/GHO/nursing-and-midwifery-personnel-(per-10-000-population)	2020/05/22
28	Licensed qualified anesthesiologists actively working (n) per 10,000 population	Total number of licensed, qualified physician anesthesiologists.	1999-2015	https://www.who.int/data/gho/data/indicators/indicator-details/GHO/number-of-licensed-qualified-	2020/05/22

				anaesthesiologists-actively-working	
29	Total expenditure on health as a percentage of GDP (%)	Level of total expenditure on health (THE) expressed as a percentage of gross domestic product (GDP).	2014	https://www.who.int/data/gho/data/indicators/indicator-details/GHO/total-expenditure-on-health-as-a-percentage-of-gross-domestic-product	2020/05/22
30	Population with household expenditures on health greater than 10% of total household expenditure or income (%)	Proportion of the population with household expenditure on health exceeding 10% of total household expenditure or income.	1993-2018	https://www.who.int/data/gho/data/indicators/indicator-details/GHO/population-with-household-expenditures-on-health-greater-than-10-of-total-household-expenditure-or-income-(sdg-3-8-2)-(-)	2020/05/22
31	Prevalence of raised blood pressure (systolic ≥140 or diastolic ≥90mmHg) (%)	Percent of defined population with raised blood pressure (systolic blood pressure ≥ 140 OR diastolic blood pressure ≥ 90).	2015	https://www.who.int/data/gho/data/indicators/indicator-details/GHO/raised-blood-pressure-(sbp-=140-or-dbp-=90)-(crude-estimate)	2020/05/22
32	Prevalence of raised fasting blood glucose (≥7.0 mmol/L or on medication) (%)	Percent of defined population with fasting glucose ≥126 mg/dl (7.0 mmol/l) or history of diagnosis with diabetes or use of insulin or oral hypoglycaemic drugs.	2014	https://www.who.int/data/gho/data/indicators/indicator-details/GHO/raised-fasting-blood-glucose-(-=-7-0-mmol-l-or-on-medication)-(crude-estimate)	2020/05/22
33	Prevalence of raised total cholesterol (≥5.0 mmol/L) (%)	Percentage of defined population with total cholesterol ≥ 190 mg/dl (5.0 mmol/l).	2008	https://www.who.int/data/gho/data/indicators/indicator-details/GHO/raised-total-cholesterol-(-=-5-0-mmol-l)-(crude-estimate)	2020/05/22
34	Mean body mass index (BMI, body weight [kg] / height^2 [m^2]	Mean body mass index (BMI) in kg/m^2 of defined population.	2016	https://www.who.int/data/gho/data/indicators/indicator-details/GHO/mean-bmi-(kg-m-)-(crude-estimate)	2020/05/22
35	Prevalence of obesity among adults, BMI ≥30 kg/m^2 (%)	Percentage of defined population with a body mass index (BMI) of 30 kg/m^2 or higher.	2016	https://www.who.int/data/gho/data/indicators/indicator-details/GHO/preval	2020/05/22

				ence-of-obesity-among-adults-bmi-=-30-(crude-estimate)-(-)	
36	Prevalence of obesity among adults, BMI ≥25 kg/m² (%)	Percentage of defined population with a body mass index (BMI) of 25 kg/m² or higher.	2016	https://www.who.int/data/gho/data/indicators/indicator-details/GHO/prevalence-of-overweight-among-adults-bmi-greaterequal-25-(crude-estimate)-(-)	2020/05/22
37	Alcohol drinking, total per capita (≥15 years of age) consumption (in liters of pure alcohol)	Total APC is defined as the total (sum of three-year average recorded and unrecorded APC, adjusted for tourist consumption) amount of alcohol consumed per adult (15+ years) over a calendar year, in liters of pure alcohol. Recorded alcohol consumption refers to official statistics (production, import, export, and sales or taxation data), while the unrecorded alcohol consumption refers to alcohol which is not taxed and is outside the usual system of governmental control. Tourist consumption takes into account tourists visiting the country and inhabitants visiting other countries. Positive figures denote alcohol consumption of outbound tourists being greater than alcohol consumption by inbound tourists, negative numbers the opposite. Tourist consumption is based on UN tourist statistics.	2016	https://www.who.int/data/gho/data/indicators/indicator-details/GHO/alcohol-total-per-capita-(15-years)-consumption-(in-litres-of-pure-alcohol)	2020/05/22
38	Prevalence of smoking any tobacco product among males aged ≥15 years (%)	The percentage of the population aged 15 years and over who currently use any tobacco product (smoked and/or smokeless tobacco) on a daily or non-daily basis. Note that most countries collect data about smoking but not smokeless tobacco use, leaving gaps in tobacco use data which prevent global and regional summaries of tobacco use rates. Until data improve, the estimates will reflect the percentage of the population aged 15 years and over who currently smoke.	2015	https://www.who.int/data/gho/data/indicators/indicator-details/GHO/prevalence-of-smoking-any-tobacco-product-among-persons-aged-=-15-years	2020/05/22
39	Prevalence of smoking any tobacco product among females aged ≥ 15 years (%)	The percentage of the population aged 15 years and over who currently use any tobacco product (smoked and/or smokeless tobacco) on a daily or non-daily basis. Note that most countries collect data about smoking but not smokeless tobacco use, leaving gaps in tobacco use data which prevent global and regional summaries of tobacco use rates. Until data improve, the estimates will reflect the percentage of the population aged 15 years and over who currently smoke.	2015	https://www.who.int/data/gho/data/indicators/indicator-details/GHO/prevalence-of-smoking-any-tobacco-product-among-persons-aged-=-15-years	2020/05/22
40	Prevalence of insufficient physical activity among adults aged ≥ 18 years (%)	Percent of defined population attaining less than 150 minutes of moderate-intensity physical activity per week, or less than 75 minutes of vigorous-intensity physical activity per week, or equivalent.	2016	https://www.who.int/data/gho/data/indicators/indicator-details/GHO/prevalence-of-insufficient-physical-activity-among-adults-aged-18-years-(crude-estimate)-(-)	2020/05/22

41	Estimated population-based prevalence of depression (%)	Number of persons with depressive disorder (major depressive disorder/depressive episode or dysthymia) in the last year. Major depressive disorder/depressive episode involves symptoms such as depressed mood, loss of interest and enjoyment, and decreased energy; depending on the number and severity of symptoms, a depressive episode can be categorized as mild, moderate, or severe	2015	https://www.who.int/data/gho/data/indicators/indicator-details/GHO/estimated-population-based-prevalence-of-depression	2020/05/22
42	Neonatal mortality rate (n per 1000 live births)	Number of deaths during the first 28 completed days of life per 1000 live births in a given year or other period. Neonatal deaths (deaths among live births during the first 28 completed days of life) may be subdivided into early neonatal deaths, occurring during the first 7 days of life, and late neonatal deaths, occurring after the 7th day but before the 28th completed day of life.	2018	https://www.who.int/data/gho/data/indicators/indicator-details/GHO/neonatal-mortality-rate-(per-1000-live-births)	2020/05/22
43	Infantile mortality rate (n per 1000 live births)	The probability of a child born in a specific year or period dying before reaching the age of five, if subject to age-specific mortality rates of that period. Under-five mortality rate as defined here is strictly speaking not a rate (i.e. the number of deaths divided by the number of populations at risk during a certain period of time) but a probability of death derived from a life table and expressed as rate per 1000 live births.	2018	https://www.who.int/data/gho/data/indicators/indicator-details/GHO/under-five-mortality-rate-(probability-of-dying-by-age-5-per-1000-live-births)	2020/05/22
44	Under-five mortality rate (probability of dying by age 5 per 1000 live births)	The probability of a child born in a specific year or period dying before reaching the age of five, if subject to age-specific mortality rates of that period. Under-five mortality rate as defined here is strictly speaking not a rate (i.e. the number of deaths divided by the number of populations at risk during a certain period of time) but a probability of death derived from a life table and expressed as rate per 1000 live births.	2018	https://www.who.int/data/gho/data/indicators/indicator-details/GHO/under-five-mortality-rate-(probability-of-dying-by-age-5-per-1000-live-births)	2020/05/22
45	Mortality rate for 5-14 years of age (probability of dying per 1000 children aged 5-14 years)	The probability that a child aged 5 dies before reaching its 15th birthday.	2018	https://www.who.int/data/gho/data/indicators/indicator-details/GHO/mortality-rate-for-5-14-year-olds-(probability-of-dying-per-1000-children-aged-5-14-years)	2020/05/22
46	Adult mortality rate (probability of dying between 15 and 60 years per 1000 population	Probability that a 15-year-old person will die before reaching his/her 60th birthday. The probability of dying between the ages of 15 and 60 years (per 1000 population) per year among a hypothetical cohort of 100,000 people that would experience the age-specific mortality rate of the reporting year.	2016	https://www.who.int/data/gho/data/indicators/indicator-details/GHO/adult-mortality-rate-(probability-of-dying-between-15-and-60-years-per-1000-population)	2020/05/22

47	Probability of dying between age 30 and exact age 70 from any of cardiovascular disease, cancer, diabetes, or chronic respiratory disease	Per cent of 30-year-old-people who would die before their 70th birthday from any of cardiovascular disease, cancer, diabetes, or chronic respiratory disease, assuming that s/he would experience current mortality rates at every age and s/he would not die from any other cause of death (e.g., injuries or HIV/AIDS).	2016	https://www.who.int/data/gho/data/indicators/indicator-details/GHO/probability-(-)-of-dying-between-age-30-and-exact-age-70-from-any-of-cardiovascular-disease-cancer-diabetes-or-chronic-respiratory-disease	2020/05/22
48	Life expectancy at birth (years)	The average number of years that a newborn could expect to live, if he or she were to pass through life exposed to the sex- and age-specific death rates prevailing at the time of his or her birth, for a specific year, in a given country, territory, or geographic area.	2016	https://www.who.int/data/gho/data/indicators/indicator-details/GHO/life-expectancy-at-birth-(years)	2020/05/22
49	Life expectancy at age 60 years (years)	The average number of years that a person of 60 years old could expect to live, if he or she were to pass through life exposed to the sex- and age-specific death rates prevailing at the time of his or her 60 years, for a specific year, in a given country, territory, or geographic area.	2016	https://www.who.int/data/gho/data/indicators/indicator-details/GHO/life-expectancy-at-age-60-(years)	2020/05/22
50	Healthy life expectancy (HALE) at birth (years)	Average number of years that a person can expect to live in "full health" by taking into account years lived in less than full health due to disease and/or injury.	2016	https://www.who.int/data/gho/data/indicators/indicator-details/GHO/healthy-life-expectancy-(hale)-at-birth-(years)	2020/05/22
51	Healthy life expectancy (HALE) at age 60 years (years)	The average number of years in full health a person (usually at age 60) can expect to live based on current rates of ill-health and mortality. Disaggregated by gender	2016	https://www.who.int/data/gho/data/indicators/indicator-details/GHO/healthy-life-expectancy-(hale)-at-age-60-(years)	2020/05/22
52	Death due to chronic obstructive pulmonary disease (%)	The mortality attributable to the joint effects of household and ambient air pollution can be expressed as: Number of deaths Death rate Death rates are calculated by dividing the number of deaths by the total population (or indicated if a different population group is used, e.g. children under 5 years). Evidence from epidemiological studies have shown that exposure to air pollution is linked, among others, to the important diseases taken into account in this estimate: Chronic obstructive pulmonary disease in adults (estimated above 25 years)	2016	https://www.who.int/data/gho/data/indicators/indicator-details/GHO/ambient-and-household-air-pollution-attributable-death-rate-(per-100-000-population)	2020/05/22

53	Death due to ischemic heart disease (%)	The mortality attributable to the joint effects of household and ambient air pollution can be expressed as: Number of deaths Death rate Death rates are calculated by dividing the number of deaths by the total population (or indicated if a different population group is used, e.g. children under 5 years). Evidence from epidemiological studies have shown that exposure to air pollution is linked, among others, to the important diseases taken into account in this estimate: Ischemic heart diseases in adults (estimated above 25 years)	2016	https://www.who.int/data/gho/data/indicators/indicator-details/GHO/ambient-and-household-air-pollution-attributable-death-rate-(per-100-000-population)	2020/05/22
54	Death due to lower respiratory infections (%)	The mortality attributable to the joint effects of household and ambient air pollution can be expressed as: Number of deaths Death rate Death rates are calculated by dividing the number of deaths by the total population (or indicated if a different population group is used, e.g. children under 5 years). Evidence from epidemiological studies have shown that exposure to air pollution is linked, among others, to the important diseases taken into account in this estimate: Acute respiratory infections (estimated for all ages)	2016	https://www.who.int/data/gho/data/indicators/indicator-details/GHO/ambient-and-household-air-pollution-attributable-death-rate-(per-100-000-population)	2020/05/22
55	Death due to stroke (%)	The mortality attributable to the joint effects of household and ambient air pollution can be expressed as: Number of deaths Death rate Death rates are calculated by dividing the number of deaths by the total population (or indicated if a different population group is used, e.g. children under 5 years). Evidence from epidemiological studies have shown that exposure to air pollution is linked, among others, to the important diseases taken into account in this estimate: Cerebrovascular diseases in adults (estimated above 25 years)	2016	https://www.who.int/data/gho/data/indicators/indicator-details/GHO/ambient-and-household-air-pollution-attributable-death-rate-(per-100-000-population)	2020/05/22
56	Death due to trachea, bronchus, lung cancers (%)	The mortality attributable to the joint effects of household and ambient air pollution can be expressed as: Number of deaths Death rate Death rates are calculated by dividing the number of deaths by the total population (or indicated if a different population group is used, e.g. children under 5 years). Evidence from epidemiological studies have shown that exposure to air pollution is linked, among others, to the important diseases taken into account in this estimate: Lung cancer in adults (estimated above 25 years).	2016	https://www.who.int/data/gho/data/indicators/indicator-details/GHO/ambient-and-household-air-pollution-attributable-death-rate-(per-100-000-population)	2020/05/22
57	Ambient and household air pollution attributable death rate (n per 100 000 population)	The mortality attributable to the joint effects of household and ambient air pollution can be expressed as: Number of deaths Death rate Death rates are calculated by dividing the number of deaths by the total population (or indicated if a different population group is used, e.g. children under 5 years). Evidence from epidemiological studies have shown that exposure to air pollution is linked, among others, to the important diseases taken into account in this estimate: Acute respiratory infections (estimated for all ages); Cerebrovascular diseases in adults (estimated above 25 years); Ischemic heart diseases in adults (estimated above 25 years); Chronic obstructive pulmonary disease in adults (estimated above 25 years); and Lung cancer in adults (estimated above 25 years).	2016	https://www.who.int/data/gho/data/indicators/indicator-details/GHO/ambient-and-household-air-pollution-attributable-death-rate-(per-100-000-population)	2020/05/22

58	Annual mean concentration of particulate matter of less than 2.5 microns of diameter ($PM_{2.5}$)	The mean annual concentration of fine suspended particles of less than 2.5 microns in diameters is a common measure of air pollution. The mean is a population-weighted average for urban population in a country.	2016	https://www.who.int/data/gho/data/indicators/indicator-details/GHO/concentrations-of-fine-particulate-matter-(pm2-5)	2020/05/22
59	Measles-containing-vaccine first dose (MCV1) immunization coverage among 1-year-olds (%)	The percentage of children under one year of age who have received at least one dose of measles-containing vaccine in a given year. For countries recommending the first dose of measles vaccine in children over 12 months of age, the indicator is calculated as the proportion of children less than 12-23 months of age receiving one dose of measles-containing vaccine.	2018	https://www.who.int/data/gho/data/themes/topics/indicator-groups/indicator-group-details/GHO/mcv1---immunization-coverage-estimates	2020/7.19
